# Cross-Species Analysis of Gene Expression and Function in Prefrontal Cortex, Hippocampus and Striatum

**DOI:** 10.1371/journal.pone.0164295

**Published:** 2016-10-07

**Authors:** Wei Chen, Xiayu Xia, Nan Song, Ying Wang, Hua Zhu, Wei Deng, Qi Kong, Xianmin Pan, Chuan Qin

**Affiliations:** 1 Institute of Laboratory Animal Science, Chinese Academy of Medical Sciences (CAMS) & Comparative Medicine Centre, Peking Union Medical Collage (PUMC), Beijing, P.R. China; 2 Ministry of Education, The Key Laboratory of Bioinformatics, School of Life Sciences, Tsinghua University, Beijing, P.R. China; University of South Florida, UNITED STATES

## Abstract

**Background:**

Mouse has been extensively used as a tool for investigating the onset and development of human neurological disorders. As a first step to construct a transgenic mouse model of human brain lesions, it is of fundamental importance to clarify the similarity and divergence of genetic background between non-diseased human and mouse brain tissues.

**Methods:**

We systematically compared, based on large scale integrated microarray data, the transcriptomes of three anatomically distinct brain regions; prefrontal cortex (PFC), hippocampus (HIP) and striatum (STR), across human and mouse. The widely used DAVID web server was used to decipher the biological functions of the highly expressed genes that were identified using a previously reported approach. Venn analysis was used to depict the overlapping ratios of the notably enriched biological process (BP) terms (one-tailed Fisher’s exact test and Benjamini correction; adjusted p < 0.01) between two brain tissues. GOSemSim, an R package, was selected to perform GO semantic similarity analysis. Next, we adjusted signal intensities of orthologous genes by the total signals in all samples within species, and used one minus Pearson’s correlation coefficient to assess the expression distance. Hierarchical clustering and principal component analysis (PCA) were selected for expression pattern analysis. Lineage specific expressed orthologous genes were identified by comparison of the most extreme sub-datasets across species and further verified using reverse transcription PCR (RT-PCR) and quantitative real-time PCR (qRT-PCR).

**Results:**

We found that the number of the significantly enriched BP terms of the highly expressed genes in human brain regions is larger than that in mouse corresponding brain regions. The mainly involved BP terms in human brain tissues associated with protein-membrane targeting and selenium metabolism are species-specific. The overlapping ratios of all the significantly enriched BP terms between any two brain tissues across species are lower than that within species, but the pairwise semantic similarities are very high between any two brain tissues from either human or mouse. Hierarchical clustering analysis shows the biological functions of the highly expressed genes in brain tissues are more consistent within species than interspecies; whereas it shows the expression patterns of orthologous genes are evidently conserved between human and mouse equivalent brain tissues. In addition, we identified four orthologous genes (*COX5B*, *WIF1*, *SLC4A10* and *PLA2G7*) that are species-specific, which have been widely studied and confirmed to be closely linked with neuro- physiological and pathological functions.

**Conclusion:**

Our study highlights the similarities and divergences in gene function and expression between human and mouse corresponding brain regions, including PFC, HIP and STR.

## Introduction

Neurological disorders have become serious threatens to human health and quality of life, especially in the low-to-middle-income countries [1,2]. To relieve this global burden, it is of fundamental importance to design an optimal animal model to explore the underlying mechanisms of such diseases with enigmatic pathogenesis. Since mouse has a small size, a short gestation period, a rich experimental history, and a mature genetic engineering technology, it has been extensively used as a tool for understanding human diseases, including brain disorders [3–5].

Although mouse’s life cycle is regarded as human’s life cycle in miniature, there are remarkable variations between human and mouse brains with respect to size, complexity, and cognitive abilities [6]. However, the successful application of numerous mouse models established to study genetic risk genes involved in neurological disorders indicates deeper biological similarities between human and mouse brains. It is crucial to know a priori whether a gene of interest is expressed or functions similarly across species when constructing a transgenic mouse model. Previous studies have paid attention to the general gene expression evolution pattern among multiple tissues, including different brain regions, between human and mouse. Some studies have demonstrated an evidently divergent expression pattern of orthologous genes [7–9], whereas some other investigations have showed that gene expression in analogous tissues (e.g., human and mouse comparable brain tissues) is highly conserved [10–12]. In addition, a recent study describes a slower transcriptome change in nervous tissues compared to other tissues among lineages [13]. So far the debate pertaining to gene expression pattern of comparable tissues between human and mouse is still in flux. Thus more evidence should be established to further clarify this issue.

Cross-species analysis of transcriptomes between healthy human and mouse brains can highlight the similarity and difference, providing a powerful approach to evaluate the effectiveness of the mouse model for human neurological diseases. Paying close attention to the species-specific expressed brain genes may also be conducive to comprehending each species typical nervous system activities or genetically susceptible brain diseases. Janssen et al. [12] found human and mouse choroid plexus epithelium (CPE) transcriptomes are highly comparable in expression and function; but there also exist a few mouse-specific CPE genes compared to human CPE, which indicates a difference in the intracranial pressure regulation and targeted vesicle transport and metabolism between the two species. Other differences include a human-specific *de novo* protein-coding gene, namely *C20orf203*, that is most abundantly expressed in brain, whose aberrant expression is involved in human-specific pathogenesis of Alzheimer’s disease (AD) [14], and *GLUD2*, a hominoid specific brain enzyme that contributes to human cognitive abilities but also appears to confer vulnerability to brain tumors [15].

Our current study mainly focused on three brain regions, PFC, HIP and STR, because these structures are anatomically distinguishable brain areas that are structurally comparable between human and mouse; each species has specific phenotypes regarding advanced emotion, language, cognitive ability and bipedalism that are closely related to these structures; and these brain structures are vulnerable targeted regions for human brain disorders, such as tumors or neurodegenerative diseases. In this study, we integrated large scale microarray data, systematically compared the biological functions of the highly expressed genes, explored the gene expression pattern and excavated species-specific expressed orthologous genes of the three corresponding brain tissues across human and mouse. We endeavor to explore the similarities and differences across human and mouse brain transcriptomes, and provide a detailed knowledge of gene expression and function for mouse modeling regarding human brain disorders.

## Materials and Methods

### Sample selection and preprocessing

Microarray data of human and mouse PFC, HIP and STR transcriptomes were downloaded from Gene Expression Omnibus (http://www.ncbi.nlm.nih.gov/geo/). Only the datasets generated from Affymetrix microarray chip platforms were considered in the present study. To reduce the intrinsic errors within Affymetrix microarray platforms, we selected the relevant chip data from two platforms of Affymetrix (for human, HG-U133A and HG-U133_Plus_2; for mouse, Mouse430A_2 and Mouse430_2). All human and mouse samples in the merged datasets were used as control groups in corresponding studies and diagnosed with no mental or neurological diseases. The basic information of these datasets is provided in **S1** (human) and **S2** (mouse) **Tables**. Primary analysis of microarray data was performed using Expression Console (Version 1.4.1, http://www.affymetrix.com/), a freely available software designed for preprocessing microarray datasets. The robust multichip average (RMA) algorithm was selected for signal intensity normalization [16]. Since RNA integrity of brain tissues is mainly affected by agonal and postmortem factors, we used the average correlation index (ACI) reported by Tomita et al.[17] to evaluate the potential perturbation of microarray expression profiles between any two brain samples within a given platform.

### Functional annotation of the highly expressed genes

The highly expressed genes are most likely of potential biological importance [12,18,19]. The strategy to discover the highly expressed genes was in accordance with previous reports [12,18,19]. Briefly, we first ranked the genes in a dataset depending on the median RMA value in ascending order and assigned percentile ranks; the ranked gene expression dataset was arbitrarily separated into four sub-datasets: “very low expression (<10%)”, “low expression (≥10% and<50%)”, “moderate expression (≥50% and <90%)” and “high expression (≥90%)”. In regard to the “high expression” sub-datasets, the shared genes between two microarray platforms within a tissue from a species (**S3 Table**) were selected for further analysis. Afterwards, we performed functional enrichment analysis (biological process, BP) using DAVID (v6.8 Beta, [20]). The statistical significance is determined using the one-tailed Fisher’s exact test followed by the Benjamini correction; adjusted p < 0.01 was considered significant.

### Functional comparison of the significantly enriched BP terms

Venn analysis was used to depict which BP terms were specific for one dataset or overlapped across two datasets. The statistical significance of overlap between two datasets was determined using the one-tailed Fisher’s exact test. A p < 0.05 indicated that the number of common BP terms shared by two different datasets was greater than what would be expected by chance. In general, the GO semantic similarity provides the basis for functional comparison of gene products. Here, we used mgoSim function from an R package, GOSemSim [21], for semantic similarity computation between two sets of BP terms. Semantic similarity was determined depending on a graph-based strategy using the topology of the GO graph structure [22]. Based on the overlapping ratios and similarity scores, hierarchical clustering with average linkage method and a Pearson distance metric was performed to separate brain tissues.

### Orthologous gene extraction and expression pattern evaluation

Human and mouse orthologous gene-pair information was obtained from Mouse Genome Informatics (MGI, http://www.informatics.jax.org/, [23]). A complete list containing 17332 pairs of human-mouse orthologs with phenotype annotations was used for orthologous gene extraction. Directly comparing gene expression data generated by different Affymetrix microarray platforms across species seems disharmonious, because different probes have different affinities for a given gene [12]. Therefore, we used an unbiased method introduced by Liao et al. [10] to measure the expression pattern of orthologous genes between corresponding human and mouse brain tissues. Briefly, first, the original signal intensity was calculated by antilog of the corresponding RMA value. Second, an adjusted signal intensity was used to represent the relative expression level of a gene in a given dataset. The formula for signal intensity adjusting was defined as Eq (1).

$$R(i,j)=S(i,j)/\sum_{j=0}^{n}S(i,j)$$ (1)

Here, *n* is the number of datasets from one species and is 6 in present study. *S(i*, *j)* indicates the antilog-transformed RMA value of gene *i* in dataset *j*. *R(i*, *j)* indicates the relative expression level of gene *i* in dataset *j*. Third, the expression divergence between any two datasets was measured by one minus Pearson’s Correlation Coefficient (1-P). P was calculated using Eq (2).

$$P=\frac{\sum_{i=1}^{N}\left[R(i,j1\right)R(i,j2)]-[\sum_{i=1}^{N}R(i,j1)][\sum_{i=1}^{N}R(i,j2)]/N}{\sqrt{\sum_{i=1}^{N}{\left[R(i,j1)\right]}^{2}-{\left(\sum_{i=1}^{N}R(i,j1)\right)}^{2}/N}\times \sqrt{\sum_{i=1}^{N}{\left[R(i,j2)\right]}^{2}-{\left(\sum_{i=1}^{N}R(i,j2)\right)}^{2}/N}}$$ (2)

Here, *N* is the total number of common orthologous genes among all the platforms. *R(i*, *j1)* or *R(i*, *j2)* represents the median value of the relative expression levels of gene *i* across all samples in dataset *j1* or *j2*. The dendrogram of datasets was derived from the hierarchical clustering algorithm implemented in R (version 3.2.2, http://www.r-project.org/) using average linkage method and “1–P” as a distance metric between two clusters. Principal components analysis (PCA) that allows identification of categorical variables (principal components, PCs) in the data based on observed variables was performed, to verify the result of hierarchical clustering. PCA was implemented using the prcomp function from the stats package in R. The confidence ellipse was drawn using the autoplot function from the ggplot2 package in R with default parameters.

### Mining of species-specific expressed orthologous genes

We cannot directly compare microarray data across two species. Thus, we adopted a methodology according to Booij et al [18] and Janssen et al [12,19]. We compared the common orthologous genes between two platforms (HG-U133A and HG-U133_Plus_2) of human “high expression” sub-datasets with the common orthologous genes between two platforms (Mouse430A_2 and Mouse430_2) of mouse “very low expression” sub-datasets. The shared orthologous genes of the four datasets were defined as human-specific expressed orthologous genes. In other words, the genes that are highly expressed in human brain regions but have very low expression in mouse corresponding brain regions are considered to be specifically expressed in human brain regions. Analogously, the mouse-specific orthologous genes expressed in brain tissues were mined using the same method, namely contrasting the two mouse “high expression” sub-datasets with the two human “very low expression” sub-datasets.

### Brain tissues and ethics statement

Human postmortem brain tissues (PFC, HIP and STR), frozen in liquid nitrogen, were obtained from the Chinese Brain Bank Center (CBBC, China, Wuhan). Informed consent for the use of human tissues for research was obtained in writing from all donors or their next of kin. All procedures of the human brain materials have been approved by the Ethics Committee of Peking Union Medical College (PUMC, Beijing, China). The basic information of these human brain tissues are listed in **S4 Table**. Eight-month-old C57BL/6 mice (n = 5, three male and two female) were purchased from Vital River Laboratories (VRL, Beijing, China) and acclimatized in cages for 3 days before sacrifice under pentobarbital (50mg/kg) anesthesia by extracting the eyeballs for bleeding accompanied by perfusion with saline. PFC (cortical piece adjacent to olfactory bulb), HIP (both sides) and STR (dorsal and ventral striatum as a tissue block) were precisely dissected according to mouse brain anatomic atlas. The specimens were immediately frozen in liquid nitrogen until use. All mice related studies were approved by the Institutional Animal Care and Use Committee of Institute of Laboratory Animal Science, Peking Union Medical College. Both of the methods for the human and mouse protocols were carried out in accordance with the approved guidelines.

### RT-PCR and qRT-PCR verification

Total RNA of each brain tissue was purified using Trizol (Invitrogen, USA) and was quantified using a spectrophotometer. The RNA samples were reverse-transcribed using PrimeScript 1^st^ Strand cDNA Synthesis Kit (Takara, Japan). Even though intrinsically shorter and partially degenerated RNA fragments usually occur in human postmortem donor materials, we designed primers near the 3’ end of a given gene in keeping with the microarray probes for RT-PCR. The densitometry values were corrected by the expression of GAPDH gene. For qRT-PCR, gene expression was quantified using an Applied Biosystem 7500 Real-Time PCR System. The assay used gene-specific primers spanning an exon-exon junction and One Step SYBR PrimeScript RT-PCR Kit (Takara, Japan), according to the manufacturer’s manual. RT-PCR and qRT-PCR were used to verify the species-specific gene expressions at the same time. All the primers used in present study are listed in **S**5 (RT-PCR) and **S6** (qRT-PCR) **Tables**.

## Results

### Integrated transcriptome microarray data of PFC, HIP and STR from human and mouse

We selected and merged a retrospective series of transcriptome profiles using public microarray data from the GEO database (for details see Materials and Methods). These profiles covered three anatomically distinct regions of non-diseased human and mouse brains, PFC (*n* = 228 for human sample, *n* = 100 for mouse sample), HIP (*n* = 134 for human sample, *n* = 102 for mouse sample) and STR (*n* = 62 for human sample, *n* = 57 for mouse sample) (**S1** and **S2 Tables**). Since the probes targeting the same sequence usually vary across different microarray products, the detection results are often nonlinear between different microarray products [24,25]. Hence, all the gene expression profiles considered in this study were generated by Affymetrix that is reported approximately linear with the actual quantity of target RNA [10]. Moreover, the most commonly used 3′-in vitro transcription (3′IVT) Affymetrix platforms (HG-U133A, HG-U133_Plus_2, Mouse430A_2, Mouse430_2) were selected. This is because 3′IVT microarrays use the oligo(dT) primer, binding to the 3′UTR region, to initiate the cDNA synthesis in the 3′->5′ direction. This achieves a very high yield of amplification in the close vicinity of the 3′ region although it is very susceptible to RNA degradation [26,27]. Only a few genes have been reported to show a significant age-related expression change in brain tissues of adult individuals [28]. Thus, all the human donors were restricted to be adults in the age range from 18 to 99; mouse samples selected in present study were also restricted to be adults (≥ 1 month). Since many factors may affect the gene expression level [11], we used ACI to evaluate the interclass variance [17]. The lowest ACI value between any two human samples from the same microarray platform is 0.799 (**S1 Table**), and the lowest ACI value between any two mouse samples from the same microarray platform is 0.868 (**S2 Table**), indicating a rather small perturbation among samples.

### Functional enrichment analysis of the highly expressed genes in human and mouse equivalent brain tissues

**Fig 1:**
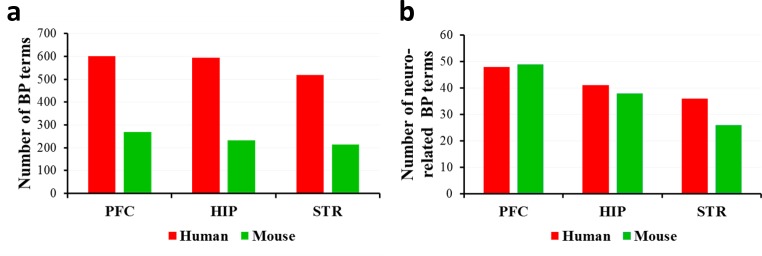
The number of the notably enriched BP terms of the highly expressed genes. The number of the significantly enriched BP terms (a) and neuro-related BP terms (b). DAVID was selected to perform functional enrichment analysis. The statistical significance is determined using the one-tailed Fisher’s exact test followed by the Benjamini correction; adjusted p < 0.01 was considered significant. Red bars represent human and blue bars mouse.

We selected the widely-used DAVID [20] to perform functional enrichment analysis of the “high expression” sub-datasets from human and mouse PFC, HIP and STR, respectively. As shown in **Fig 1A**, the number of the significantly enriched BP terms (adjusted p < 0.01) for human brain tissues is observed to be over two times than that for mouse brain tissues (detailed information is provided in **S7 Table**). However, the number of the evidently enriched neuro-related BP terms (adjusted p < 0.01) appears to show no variation between human and mouse corresponding brain regions (**Fig 1B**, detailed information is provided in **S8 Table**). Even so, several neuro-related BP terms, such as “central nervous system development”, “midbrain development”, “brain development”, “neuron projection guidance”, “axon guidance”, “neural nucleus development”, “head development”, “locomotion”, and “learning”, are specific to human, in comparison with mouse (**S8 Table**). Next, we ranked all BP terms according to Benjamini-corrected p values in descending order. The most significantly enriched (top ten) BP terms in mouse brain tissues are also found in human brain tissues, whereas the top ten BP terms in human brain tissues pertaining to protein-membrane targeting and selenium metabolism are found to be species-specific with respect to mouse (**S9 Table**).

### Functional comparison of the highly expressed genes in human and mouse analogous brain tissues

**Fig 2:**
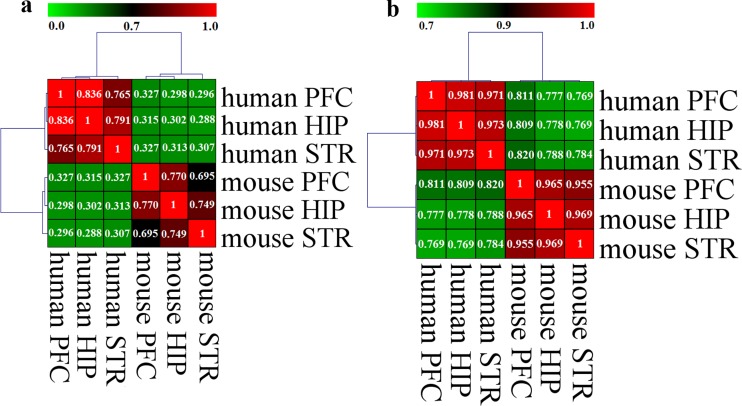
Overlapping ratio and semantic similarity analyses. (a) Comparison of the overlapping ratios. The overlapping ratio was defined as the number of the intersection divided by the number of the union of two BP term sets. The overlap significance was determined using the one-tailed Fisher’s exact test. Hierarchical clustering with a Pearson distance metric and average linkage method was performed based on the overlapping ratios. (b) Comparison of the semantic similarities. Semantic similarity between two sets of BP terms was measured using mgoSim function from an R package termed GOSemSim. The method parameter was set as “Wang” and the GO used in measurement was restricted by assigning the corresponding parameter to ‘BP’. The similarity score is between 0 and 1. The higher the score, the more similarity between two sets of BP terms. Hierarchical clustering with a Pearson distance metric and average linkage method was performed based on the calculated similarity scores.

The highly expressed genes of the analogous brain tissues across species were enriched in both species-specific and shared BP terms. The overlapping ratios of the significantly enriched BP terms are 32.7%, 30.2% and 30.7%, respectively, between human and mouse PFC, HIP and STR (**Fig 2A**). However, the smallest overlapping ratio of the evidently enriched BP terms between any two brain tissues within species is 69.5%, which is approximately twofold larger than any one across species (**Fig 2A**). In all cases, the agreement between any two brain regions is greater than expected by chance (Fisher’s exact test, p < 2.2 x 10^−16^). Furthermore, we performed semantic similarity analysis of the notably enriched BP terms between any two brain tissues using GOSemSim. Semantic similarity can be, to some extent, used for evaluating functional coherence of different sets of genes [21]. The smallest similarity score between any two brain tissues within species is 0.971, which is greater than the highest similarity score (0.820) between any two brain tissues across species (**Fig 2B**). Based on the overlapping ratios or semantic similarity scores, hierarchical clustering with a Pearson distance metric and average linkage method significantly separates the brain tissues into two clusters corresponding to the two species, human and mouse, implying a more species-dominated similarity (**Fig 2A** and **2B**).

### Globally conserved gene expression pattern between human and mouse equivalent brain structures

**Fig 3:**
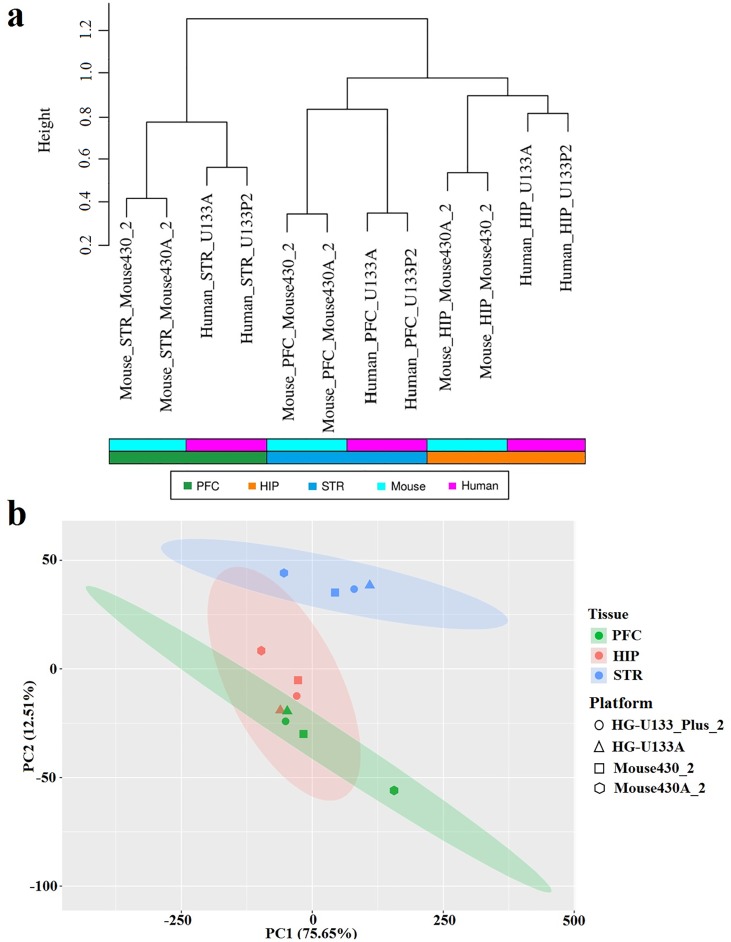
Gene expression pattern analysis. (a) Hierarchical clustering dendrogram of the 12 datasets (distance metric: “1-P”, linkage method: complete). The color bars below the dendrogram provide information about the species (upper bar) and the datasets (lower bar). (b) Principal component analysis (PCA) of the 12 datasets. Brain tissues are labeled by different colors (green = PFC; red = HIP; blue = STR). Microarray platforms are labeled by different shapes (circle = HG-U133_Plus_2; triangle = HG-U133A; square = Mouse430_2; hexagon = Mouse430A_2). The ellipse shows 95% confidence intervals.

After orthologous gene mining, as shown in **S1A Fig**, we found 12150, 17195, 12510 and 16687 orthologous genes in HG-U133A, HG-U133_Plus_2, Mouse430A_2 and Mouse430_2 platforms, respectively. For further gene expression similarity analysis, the common orthologous genes (n = 8659) among the four microarray platforms were used. These genes expression is observed similar overall across all brain tissue samples (**S1B Fig**). We normalized the signal intensities of the common orthologous genes using the total signals in all datasets within species. The expression distance between any two datasets was assessed by “1-P” (see Materials and Methods). The dendrogram derived from hierarchical clustering (**Fig 3A**) shows that Affymetrix chip platforms measuring one brain tissue from one species tend to be markedly clustered, indicating a high degree of consistency although some Affymetrix probes with name suffixes _x_at and _s_at are deemed to be prone to cross-hybridization [29,30]. Analogous brain tissues across species are inclined to be evidently more clustered than brain tissues within species or non-analogous brain tissues across species (**Fig 3A**). This implies a conserved gene expression pattern between comparable human and mouse brain tissues. The PCA, however, is less clear in this regard (**Fig 3B**). We applied the 8659 orthologous genes that were considered as observed variables to PCA and used the categorical variables (median value of the coefficients of the linear combinations of the observed variables among all samples within a dataset) to visualize the PCs later. The first two PCs that are the most informative explain ~75.7% and ~12.5%, respectively, of the total observed variances. While the first two PCs clearly distinguish STR from PFC and HIP, they does not distinctly separate PFC from HIP; PFC and HIP tend to cluster close together (**Fig 3B**).

### Species-specific expressed orthologous genes in brain tissues

Comparing human “high expression” datasets with mouse “very low expression” datasets in terms of the analogous brain tissues, as shown in **S2A Fig**, we found *NSA2* is highly expressed in all three brain tissues of human but almost undetectable in mouse brain tissues; *COX5B* is highly expressed in human PFC but lowly expressed in mouse PFC; *PEG10* and *WIF1* are highly expressed in human STR but lowly expressed in mouse STR. In the same way, by comparing mouse “high expression” datasets with human “very low expression” datasets in terms of the analogous brain tissues, as shown in **S2B Fig**, we found *SLC4A10* is highly expressed in all three brain tissues of mouse but lowly expressed in human corresponding brain regions; *PLA2G7* is highly expressed in mouse HIP but lowly expressed in human HIP. Since there exist intrinsic demerits for microarray products, such as cross-hybridization and low sensitivity, the species-specific expressed genes will be further verified.

### Verification of gene expression by RT-PCR and qRT-PCR

**Fig 4:**
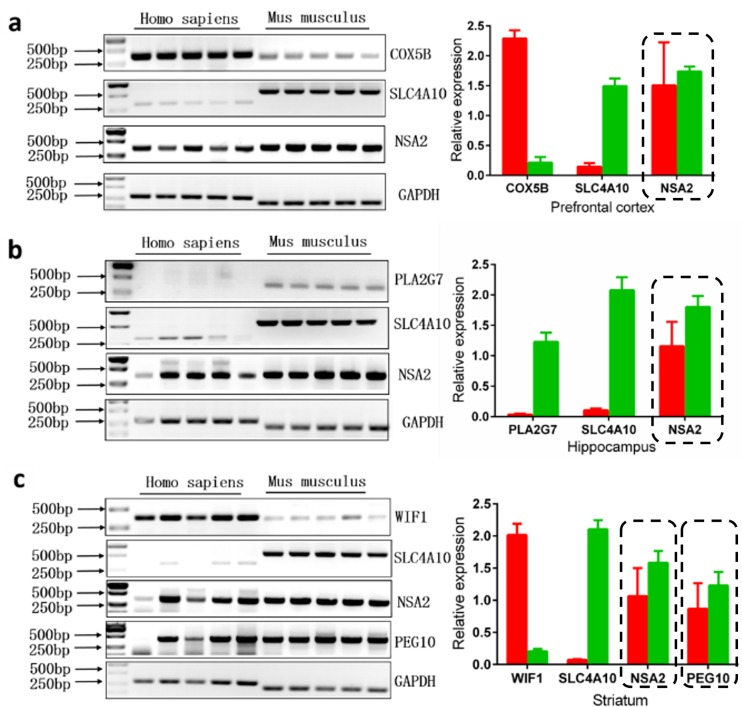
Confirmation of Affymetrix microarray data by RT-PCR. *GAPDH*, a housekeeping gene, was used to normalize gene expression in human and mouse (a) PFC, (b) HIP and (c) STR. Genes that are predicted by miacroarray data but not verified by RT-PCR are highlighted using dashed boxes. The background and band colors were reversed. The average relative densitometry values were analyzed using Image J software. Data are expressed as mean ± SD. Red bars represent human and green bars mouse.

**Fig 5:**
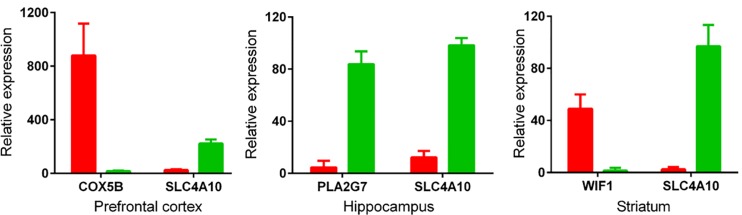
Confirmation of Affymetrix chip data by qRT-PCR. GAPDH was used to normalize gene expression. The relative expression of each gene was calculated as log2 of 2^-△Ct^ values. Data are expressed as mean ± SD. Red bars represent human and green bars mouse.

To confirm the expressions of species-specific expressed orthologous genes in brain tissues predicted by comparing the most extreme microarray sub-datasets across human and mouse, we first performed RT-PCR. As shown in **Fig 4A–4C**, *COX5B* and *WIF1* are highly expressed in human PFC and STR, respectively; *SLC4A10* is highly expressed in almost all the mouse brain tissues, encompassing PFC, HIP and STR; *PLA2G7* is highly expressed in mouse HIP compared to the comparable human HIP. However, *NSA2* and *PEG10* that are identified as specifically expressed orthologous genes in human brain tissues are also highly expressed in mouse corresponding brain tissues. To further validate these results, we designed intron-spanning primers to perform qRT-PCR. The result is in accordance with that of RT-PCR in terms of the following four genes, *COX5B*, *SLC4A10*, *PLA2G7* and *WIF1* (**Fig 5**).

## Discussion

Mouse is a facile and low-cost species for genetic modification studies compared to other laboratory animals [3–5]. Brain anatomical structure and cell type within comparable brain regions across human and mouse are largely conserved [11]. Nevertheless, neuronal behaviors, such as learning and memory, language, or tool making, are quite different in mouse from human. The number of neurons and neuronal connections in human is much larger than that in mouse, indicating a discrepancy in the complexity of neuroanatomy [6,11]. Previous studies showed that the highly expressed genes are most likely of potential biological importance [12,18,19]. In the current study, we found the total number of the related BP terms of the highly expressed genes in human brain regions is larger than that in mouse corresponding brain regions; the mainly involved BP terms in human brain tissues regarding protein-membrane targeting and selenium metabolism are species-specific, which may provide promising clues for comprehending the differences in phenotypes and responses to conditions in human and mouse. In addition, hierarchical clustering, based on either overlapping ratios or GO semantic similarity scores, reveals a more species-dominated similarity in biological function. The gene expression profiles considered consist of numerous samples coming from different studies, which partly can reduce the relative bias caused by the mortem delay, tissue dissection and gender ratio of the samples, as well as the different probe affinities. Our approach to obtain the highly expressed genes has its limitations, for example, the criteria for high and low expression levels are arbitrary. In general, orthologous genes are assumed to retain equivalent functions in different organisms and to share other key properties [31]. However, previous studies have showed that non-conservatively expressed orthologous protein-encoding or non-coding genes are involved in brain evolution and function [11,32]. Hence, the different orthologous genes in the highly expressed gene sets may be a major force driving functional variations.

In accordance with some previous studies [10–12], our present study also confirms an overall conserved expression pattern of orthologous genes between human and mouse corresponding brain regions using a hierarchical clustering algorithm. Although PCA analysis distinctly separates STR from PFC and HIP, the latter two still tend to cluster close together. Generally speaking, the choice of the similarity measure can have an effect on the result of hierarchical clustering algorithm. In this study, we used one minus Pearson’s correlation as a distance metric. Pearson correlation has been reported to be less susceptible to noise than other similarity measures [33]. The aim of the hierarchical clustering algorithm is to divide the objects into homogeneous groups, such that the within-group similarities are large compared to the between-group similarities. The PCs, on the other hand, are extracted to represent the patterns encoding the highest variance in the data set and not to maximize the separation between groups of samples directly. When using PCA to approximate a data matrix, the fraction of the total variance in the leading PCs is used as a criterion for choosing how many of them to use [33]. However, the first PCs do not necessarily capture most of the cluster structure [34]. Clustering with the PCs instead of the original variables does not necessarily improve but often degrades cluster quality [34]. In addition, to some extent, the true conservation of the gene expression pattern across human and mouse equivalent brain regions may be underestimated. The possible reasons have been discussed in detail in [11]. Briefly, these factors, such as the age, gender, anatomical position of the samples, the technical variability, and the ever-changing probe sequences, were not strictly controlled. Our result is in disagreement with some other studies [7–9]. We suspect that direct comparison of absolute signal intensity or log-transformed signal intensity of orthologous genes between human and mouse may be a major cause for disagreement with our study. In this study, we compared the relative expression levels of orthologous genes corrected by the total signals in all samples within species, which can reduce the background noise. Moreover, 8659 human-mouse orthologous gene pairs were analyzed in our study, which is sixfold more than other studies, implying the small number of orthologous gene pairs may yield a bias result [7,8].

Due to different affinities of probes to target RNAs across species, it is difficult to interpret the variations by directly comparing human and mouse microarray data. Thus, we compared the most extreme sub-datasets across species (see Materials and Methods). The orthologous genes highly expressed in human brain tissues but with low expression in corresponding mouse brain tissues, or vice versa, are defined as the species-specific expressed genes, which may have important implications in functional variations. Although a cluster of such genes were examined by this strategy, two (*NSA2* and *PEG10*) were confirmed to be false positive by RT-PCR. This may have been caused by significantly different probe affinities for a given gene across species. Brain fractured RNA segments are usually associated with agonal and postmortem factors [17,35]. Also, the microarray platforms involved here are 3′IVT microarrays that are very susceptible to RNA degradation [26,27]. Therefore, qRT-PCR using primers spanning an exon-exon junction may have further ensured the accuracy of the results.

The species-specific expressed orthologous genes detected in our study have been extensively studied in previous studies (**Table 1**). *COX5B* is a component of cytochrome c oxidase (complex IV) that is responsible for electron transport. Complex IV activation can contribute to sustaining a reduced reactive oxygen species production [36,37], an enhanced resilience to stress [38], and a continuous antioxidant defense [39]. Decreasing expression of *COX5B* will lead to a failure in complex IV assembly [40], subsequently causing brain mitochondria dysfunction which has been reported to be associated with some neurodegenerative disorders of the central nervous system, such as multiple sclerosis (MS) [41–44], spinobulbar muscular atrophy (SBMA) [45], Alzheimer’s disease (AD) [46–48] and Parkinson’s disease (PD) [49,50]. *WIF1* acting as a Wnt antagonist and tumor suppressor is involved in the pathogenesis of some brain cancers, such as astrocytomas [51–53], glioblastoma [54,55] and neuroblastoma [56]. Re-expression of *WIF1* in glioblastoma inhibits migration, suggesting a role of *WIF1* in the regulation of cell cycle and proliferation [54,55]. In addition, *WIF1* has also been confirmed to be directly implicated in the myelination process and hippocampal development, abnormal expression of which may be a possible risk for cognitive defect and dementia [57,58]. *SLC4A10*, encoding a Na^+^-dependent Cl^-^–HCO3^-^ exchanger, is a major contributor to physiological cerebrospinal fluid (CSF) secretion [59]. CSF production and turnover have been validated helpful for clearing toxic Aβ from the interstitial-fluid space of the brain to the bloodstream [60]. Diminished CSF formation elicited by abnormal expression of *SLC4A10*, with subsequently reduced ability of Aβ, is suspected to be a risk factor for AD onset and progression [60–62]. Furthermore, *SLC4A10*, by mediating acid extrusion, can also regulate intracellular pH, while acid-base homeostasis in the central nervous system is vital to neuronal excitability [59,63]. *SLC4A10* is highly expressed in mouse brain but poorly expressed in human brain, which indicates a different basal neuronal excitability and regulation. Disruption of *SLC4A10* gene in mice does not exert obvious behavioral abnormalities, but yields compromised regulation of neuronal pH and an ascending seizure threshold [59]. *SLC4A10* is widely reported as a candidate risk-related gene for some neuro-related disorders linked with neuronal excitability, such as epilepsy, autism and major depressive disorder (MDD) [59,64–66]. *PLA2G7*, encoding for platelet-activating factor acetylhydrolase (PAF-AH), promotes the degradation of PAF, generating the biologically inactive products lyso-platelet activating factor (lyso-PAF). Abnormally expressed *PLA2G7* may give rise to dysregulation of phospholipid metabolism. *PLA2G7* gene has been widely studied as a candidate risk factor of coronary heart disease [67,68]. Only a few reports endeavored to explore the potential roles of *PLA2G7* in neurophysiological processes and pathological disorders. Meng et al. [69] found *PLA2G7* may affect the clinical manifestation of schizophrenia. A meta-analysis based on differentially expressed genes in autism identified *PLA2G7* as a genetic marker involved in the development and progression of child autism [70]. Overall, it is more likely that the four species-specific orthologous genes have irreplaceable biological functions and aberrant expressions may be linked with relevant brain disorders. However, the causal relationships between these genes and brain disorders are not very clear and remain to be further established.

**Table 1:** Neurobiological roles of species-specific expressed orthologous genes.

Gene symbol	Description	Expression ^a^	Physiological processes ^b^	Diseases ^c^
*COX5B*	cytochrome c oxidase subunit Vb	Human PFC	1. reactive oxygen species 2. production resilience to stress 3. antioxidant defense	1. MS 2. SBMA 3. AD 4. PD
*WIF1*	WNT inhibitory factor 1	Human STR	1. tumor suppressor 2. myelination process 3. hippocampal development	1. astrocytomas 2. glioblastoma 3. neuroblastoma 4. dementia
*SLC4A10*	solute carrier family 4, sodium bicarbonate	Mouse PFC	1. CSF secretion 2. neuronal excitability	1. AD 2. Epilepsy 3. Autism 4. MDD
		Mouse HIP		
		Mouse STR		
*PLA2G7*	Phospholipase A2 group VII	Mouse HIP	1. phospholipid metabolism	1. Schizophrenia 2. child autism

^a^ The species and brain regions that the identified orthologous genes are highly expressed in.

^b^ The relevant physiological processes that the species-specific expressed orthologous genes are involved in.

^c^ The associated diseases caused by the dysfunctions of the corresponding species-specific genes.

Abbreviations: PFC = prefrontal cortex; HIP = hippocampus; STR = striatum; CSF = cerebrospinal fluid; MS = multiple sclerosis; SBMA = spinobulbar muscular atrophy; AD = Alzheimer’s disease; PD = Parkinson’s disease.

In summary, we systematically integrated and analyzed large scale microarray data of human and mouse PFC, HIP and STR, and confirmed a more species-dominated similarity in biological function and a conserved expression pattern of orthologous genes between the equivalent brain structures across human and mouse. We also identified a cluster of species-specific expressed orthologous genes by both microarray data and experimental verification. These genes have been widely studied and found to be closely linked with multiple neurophysiological and pathological processes. More attentions should be paid to these four species-specific orthologous genes in future studies.

## Supporting Information

S1 Fig Common orthologous genes and their expressions. (a) Venn diagram depicts the number of the common orthologous genes among four Affymetrix microarray platforms that are labeled by different colors. GN represents gene number. (b) Heatmap of expression for the common orthologous genes across all the samples. Before heatmap generation, the RMA values of all the genes were ranked in a descending order according to the average values across all the samples. The three bars above the heatmap represent species, brain tissues and microarray platforms, respectively, that are labeled by different colors from top to bottom. Pink = human, light blue = mouse, green = PFC, red = HIP, blue = STR, black = HG-U133A or Mouse430A_2, and white = HG-U133_Plus_2 or Mouse430_2.
(TIF)

S2 Fig Species-specific expressed orthologous genes in brain tissues. (a) Human- or (b) mouse-specific expressed orthologous genes in brain tissues are located in the overlap of the four datasets. Our focus is limited to the genes located in the overlap shared by the four datasets to obtain more precise results.
(TIF)

S1 Table Basic information of gene expression profiles from human regional brain tissues. (XLSX)

S2 Table Basic information of gene expression profiles from mouse regional brain tissues. (XLSX)

S3 Table The highly expressed genes in PFC, HIP and STR from human and mouse. (XLSX)

S4 Table Basic information of human brain tissues used for RT-PCR and qRT-PCR verification. (XLSX)

S5 Table Primers for RT-PCR. (DOCX)

S6 Table Primers for qRT-PCR. (DOCX)

S7 Table Significantly enriched BP terms of the highly expressed genes in human and mouse equivalent brain tissues. (XLSX)

S8 Table Significantly enriched neuro-related BP terms of the highly expressed genes in human and mouse equivalent brain tissues. (XLSX)

S9 Table The most significantly enriched (top ten) BP terms of the highly expressed genes in human and mouse brain tissues. (XLSX)
