# Supplementary figures and images for: Cross-Species Analysis of Gene Expression and Function in Prefrontal Cortex, Hippocampus and Striatum
Source: PLoS One. 2016 Oct 7;11(10):e0164295. doi: 10.1371/journal.pone.0164295 (PMC5055290; doi:10.1371/journal.pone.0164295)

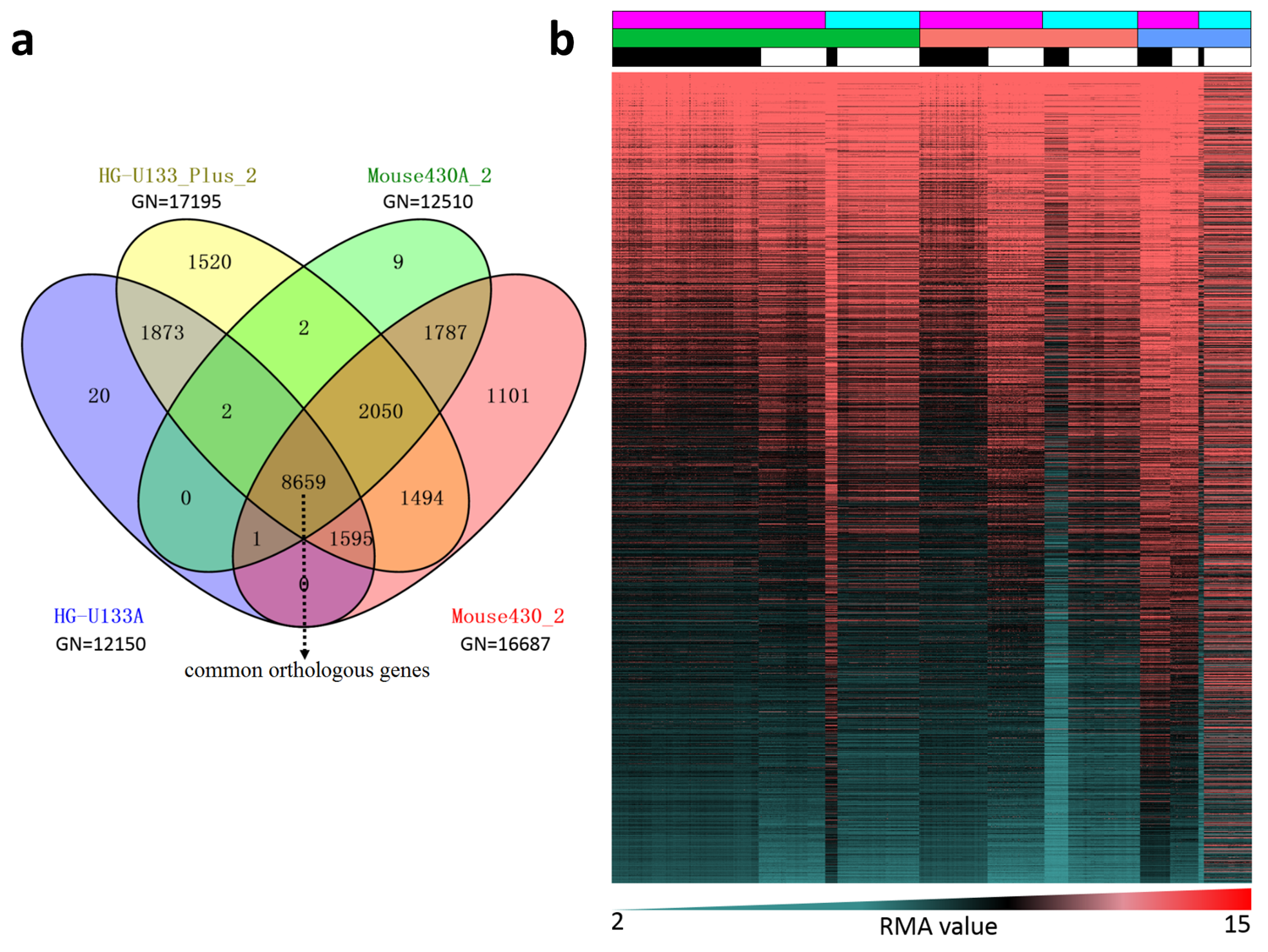

Supplement: S1 Fig — (a) Venn diagram depicts the number of the common orthologous genes among four Affymetrix microarray platforms that are labeled by different colors. GN represents gene number. (b) Heatmap of expression for the common orthologous genes across all the samples. Before heatmap generation, the RMA values of all the genes were ranked in a descending order according to the average values across all the samples. The three bars above the heatmap represent species, brain tissues and microarray platforms, respectively, that are labeled by different colors from top to bottom. Pink = human, light blue = mouse, green = PFC, red = HIP, blue = STR, black = HG-U133A or Mouse430A_2, and white = HG-U133_Plus_2 or Mouse430_2. (TIF) [file pone.0164295.s001.tif]

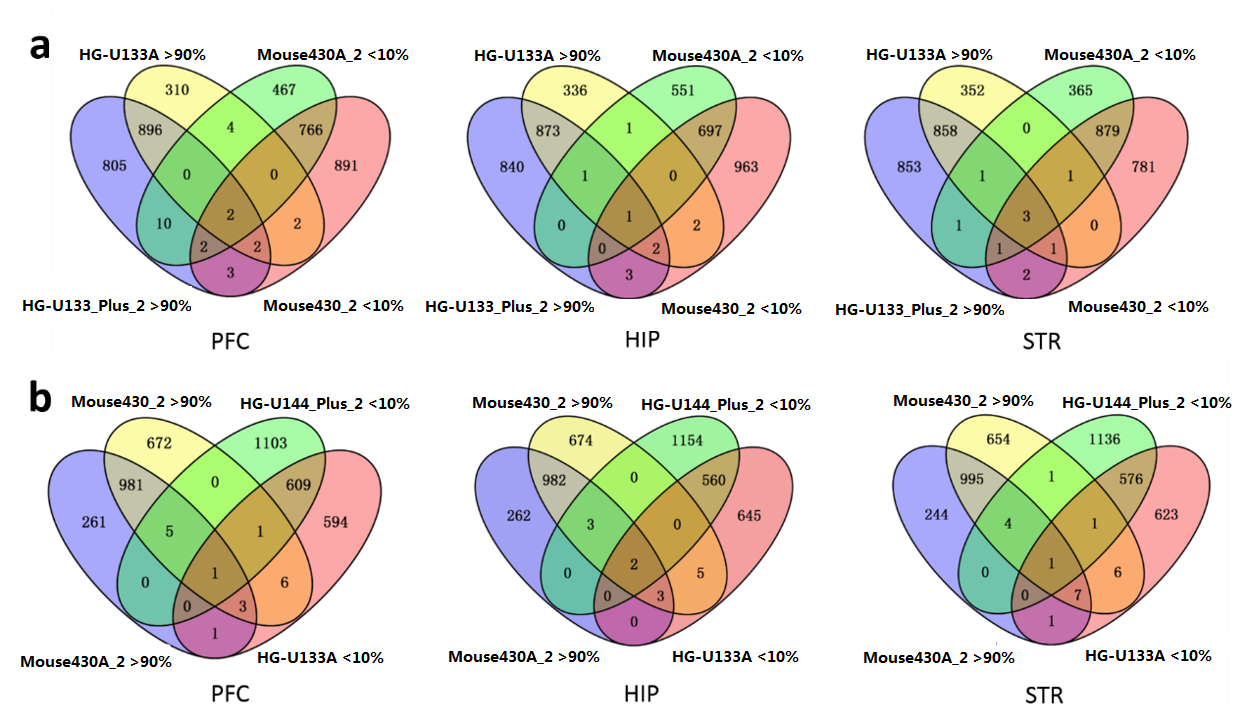

Supplement: S2 Fig — (a) Human- or (b) mouse-specific expressed orthologous genes in brain tissues are located in the overlap of the four datasets. Our focus is limited to the genes located in the overlap shared by the four datasets to obtain more precise results. (TIF) [file pone.0164295.s002.tif]
