# Supplementary material for: Cross-Species Analysis of Gene Expression and Function in Prefrontal Cortex, Hippocampus and Striatum
Source: PLoS One. 2016 Oct 7;11(10):e0164295. doi: 10.1371/journal.pone.0164295 (PMC5055290; doi:10.1371/journal.pone.0164295)
Supplement: S5 Table — (DOCX) [file pone.0164295.s007.docx]

**S5 Table. Primers for RT-PCR.**

| Gene^a^ | Sense sequence | Antisense sequence | Length | Ta Opt^b^ |
| --- | --- | --- | --- | --- |
| NSA2 | ATTGGTCTGAAGGCTAAG | TTTCTTGTAAAGCCATCTC | 362 | 51 |
| Nsa2 | TGAAAGTTATTCGGACAGG | ACCAGCAAGACCGCATTT | 382 | 51 |
| COX5B | GTGTTCCCACTGATGAAGAGC | GGAGAAGGAGCCAATGCA | 366 | 57 |
| Cox5b | TCTTGCTCAGCCTGTTCC | GATGCAGCCCACTATTCTC | 323 | 57 |
| PEG10 | TAAAGAAGTTAGAAAGGGAG | AGATGTCTGTGGTGGATG | 455 | 49 |
| Peg10 | CAACAAGTGGACGAGTG | GGTTAGGGAGTGGAAGG | 418 | 49 |
| WIF1 | CTGGTGAACTCCGACAT | AAATTCTTGTGACTTACGC | 378 | 48 |
| Wif1 | TCTAAGCGTGTCTAGCATG | AGAGTAACAGCAAGGGTGA | 360 | 48 |
| PLA2G7 | AGCAACGGTTATTCAGA | CAATCAAGCAGTCCCAC | 401 | 49 |
| Pla2g7 | GTTCTTCAAGCCCTTAG | CATCATCTCCTTCCACC | 406 | 49 |
| SLC4A10 | TACTGCCGATAACTCAAA | CTCACAGGGATGTCTCAC | 336 | 50 |
| Slc4a10 | GCCAGCCAAACATCAAC | AAGTCTCCCTGTCAACG | 512 | 50 |
| GAPDH | GGGAAACTGTGGCGTGAT | GGGTGTCGCTGTTGAAGT | 295 | 56 |
| Gapdh | GGGCATCTTGGGCTACAC | TCCAGGGTTTCTTACTCCTT | 207 | 56 |

^a^ Primers were designed using Primer Primer 5 software. Human gene symbols are written by uppercase letters; mouse gene symbols are written beginning with an uppercase letter.

^b^ Ta Opt indicates annealing temperature.
