# Supplementary material for: Cross-Species Analysis of Gene Expression and Function in Prefrontal Cortex, Hippocampus and Striatum
Source: PLoS One. 2016 Oct 7;11(10):e0164295. doi: 10.1371/journal.pone.0164295 (PMC5055290; doi:10.1371/journal.pone.0164295)
Supplement: S6 Table — (DOCX) [file pone.0164295.s008.docx]

**S6 Table. Primers for qRT-PCR.**

| Gene^a^ | Sense sequence | Antisense sequence | Length | Ta Opt^b^ |
| --- | --- | --- | --- | --- |
| COX5B | GGCTGCATCTGTGAAGAGGAC | AGCTTGTAATGGGCTCCACA | 98 | 60 |
| Cox5b | GACCGGGCGTTGTTAGACT | CCCGAGACAAGCAGATGCAC | 85 | 60 |
| WIF1 | TGTTCAAAGCCTGTCTGCGA | GAGGCTGGCTTCGTACCTTT | 123 | 60 |
| Wif1 | CCTCACAAGGCATCAGTTGTTC | TGGCATTCTGAGGGGTCCTA | 133 | 60 |
| PLA2G7 | GCATCAGGTCTGCGGAAAGG | TTTCAGCTTAGTCTCCTGGACC | 108 | 60 |
| Pla2g7 | CGGAGCCTTCAGGACGATTT | TCTCTGTGTTCGACAGTGGC | 84 | 60 |
| SLC4A10 | TACTAAGCAGAGCGAGTGCC | TCATCATTTCTCGTAGGCAGCA | 144 | 60 |
| Slc4a10 | GACACTACAGAGACGACCCG | CTCCTTTCGGCTTTCATTGCT | 146 | 60 |
| GAPDH | GAAAGCCTGCCGGTGACTAA | GCCCAATACGACCAAATCAGAG | 150 | 60 |
| Gapdh | GGAGAGTGTTTCCTCGTCCC | ATGAAGGGGTCGTTGATGGC | 136 | 60 |

^a^ Primers were designed using web-based Primer-BLAST software (http://www.ncbi.nlm.nih.gov/tools/primer-blast/). Human gene symbols are written by uppercase letters; mouse gene symbols are written beginning with an uppercase letter.

^b^ Ta Opt indicates annealing temperature.
